# Comparison of Neutrophil-to-Monocyte-Plus-Lymphocyte Ratio and Systemic Immune-Inflammation Index Between Pulmonary Tuberculosis and Community-Acquired Pneumonia Patients

**DOI:** 10.7759/cureus.117818

**Published:** 2026-10-12

**Authors:** Arie G Nugroho, I K Suryana, Ni Luh Putu E Arisanti, Ida Ayu Jasminarti D Kusumawardani, Ni W Candrawati, I Gede K Sajinadiyasa

**Affiliations:** 1 Pulmonology Department, Udayana University, Denpasar, IDN; 2 Internal Medicine Department, Udayana University, Denpasar, IDN

**Keywords:** community-acquired pneumonia, inflammation, nmlr, pulmonary tuberculosis, sii

## Abstract

Introduction: Pulmonary tuberculosis (PTB) and community-acquired pneumonia (CAP) are significant global health challenges with overlapping clinical manifestations, which often complicates rapid diagnosis in resource-limited settings. This study evaluates the potential of the neutrophil-to-monocyte-plus-lymphocyte ratio (NMLR) and the systemic immune-inflammation index (SII) as affordable and accessible hematological markers to help differentiate between these two respiratory infections.

Methods: A retrospective, cross-sectional observational study was conducted at Ngoerah Hospital, Denpasar, Indonesia, using medical record data from January 2022 to September 2025. The subjects included 25 PTB patients and 24 CAP patients who met the eligibility criteria. Median values of NMLR and SII were compared using the Mann-Whitney U test.

Results: CAP patients were generally older (mean age 61.0 years) and had a higher male prevalence (75%) compared to PTB patients (mean age 47.4 years; 44% male). All CAP patients (100%) presented with comorbidities, while TB patients showed a lower prevalence of 64%. Although the CAP group exhibited higher median values for NMLR (3.26 vs. 2.63; p=0.082) and SII (1335.77 vs. 1241.45; p=0.280), these differences did not reach statistical significance.

Conclusion: No significant differences were found in NMLR and SII values between PTB and CAP patients in the study. This suggests that a high burden of chronic comorbidities, such as heart failure and chronic kidney disease, may mask specific host immune responses to acute and chronic infections. This study stresses the limitations of single inflammatory indices in populations with multiple comorbidities, emphasizing that further investigation into multiparametric diagnostic models is vital for enhancing management precision.

## Introduction

Pulmonary tuberculosis (PTB) and community-acquired pneumonia (CAP) continue to be major infectious diseases worldwide, especially in Indonesia. Differentiating PTB from CAP at the initial presentation can be challenging because both conditions may present with similar symptoms, including cough, fever, dyspnea, fatigue, and weight loss. Early identification of PTB is particularly important because delayed diagnosis may prolong transmission and postpone appropriate anti-tuberculosis treatment. Microbiological confirmation of PTB can be obtained using mycobacterial culture or molecular assays such as Xpert MTB/RIF. However, the availability and turnaround time of these diagnostic tests may vary between healthcare facilities, particularly in settings with limited resources [1,2].

Complete blood count (CBC) is routinely available in clinical practice and provides measurements of neutrophils, monocytes, lymphocytes, and platelets. These parameters can be combined into several hematological indices, including the neutrophil-to-monocyte-plus-lymphocyte ratio (NMLR) and the systemic immune-inflammation index (SII). Both indices have been investigated as markers of the systemic inflammatory response. In CAP, acute bacterial infection is generally associated with neutrophil-predominant inflammation, whereas PTB produces a more prolonged immune response related to the interaction between *Mycobacterium tuberculosis* and host immune cells. This difference in the inflammatory response may be reflected in peripheral blood cell counts and derived inflammatory indices. Previous studies have reported that NMLR and SII may differ between patients with PTB and those with other pulmonary infections, although the reported diagnostic performance and cutoff values have varied among study populations [3-5].

The diagnostic value of these inflammatory indices may also be influenced by factors unrelated to the underlying pulmonary infection. This is particularly relevant in tertiary referral hospitals, where patients frequently have concomitant chronic diseases such as chronic kidney disease, heart failure, diabetes mellitus, and hypertension. These conditions may alter peripheral blood cell counts and contribute to chronic systemic inflammation, potentially affecting the interpretation of NMLR and SII. Therefore, evaluating these indices in a tertiary care population with a substantial comorbidity burden may provide a more clinically relevant assessment of their diagnostic utility [6,7].

This retrospective, cross-sectional observational study evaluated pre-treatment medical records of patients admitted to Ngoerah Hospital between January 2022 and September 2025. The study included 25 treatment-naive patients with bacteriologically confirmed PTB and 24 treatment-naive patients with CAP. The primary objective of this study was to compare peripheral blood NMLR and SII values between patients with PTB and CAP. As a secondary exploratory objective, we examined the associations of categorized NMLR and SII values with PTB and CAP diagnoses using previously reported literature-based and sample-derived cutoffs, including multivariable analyses accounting for potential confounding factors.

## Materials and methods

This was a retrospective, cross-sectional observational study using medical record data. Data extraction was conducted at Ngoerah Hospital from October 2025 to December 2025 using medical records of patients admitted between January 2022 and September 2025. The target population consisted of adult patients with PTB or CAP, while the accessible population consisted of PTB and CAP patients hospitalized at Ngoerah Hospital during the study period. Eligible patients were selected consecutively until the minimum required sample size was achieved.

The sample size was calculated based on the expected between-group difference in SII reported in previous studies, using the cross-sectional formula described by Machin et al., with a 95% confidence level ($\mathrm{Z}_{1-&alpha;/2}=1.96$), 80% statistical power ($\mathrm{Z}_{1-&beta;}=0.84$), an expected mean difference (*&Delta;*) of 1560.52, and standard deviations of 2704 and 164.68 obtained from previous studies [8-10]. The calculation resulted in a minimum sample size of 24 subjects for each group. The final sample consisted of 49 eligible patients, including 25 treatment-naive patients with bacteriologically confirmed PTB and 24 treatment-naive patients with CAP.

The inclusion criteria were patients aged ≥18 years who met the diagnostic criteria for either treatment-naive PTB or treatment-naive CAP. PTB was confirmed bacteriologically using GeneXpert MTB/RIF or mycobacterial culture [11]. CAP was diagnosed based on the presence of at least two acute clinical features, including fever (>38°C or <36°C), leukocytosis (>10,000/µL) or leukopenia (<4,000/µL), cough, purulent sputum, chest pain, dyspnea, or signs of pulmonary consolidation, together with a new pulmonary infiltrate or air bronchogram on chest imaging [12]. The diagnoses were verified by two attending pulmonologists based on the documented clinical, radiological, and microbiological findings, as applicable. Treatment-naive status indicated that patients had not received disease-specific treatment before baseline laboratory assessment.

Patients were excluded if their medical records were incomplete or if they had an underlying primary hematological disorder, secondary immunodeficiency, pregnancy, extrapulmonary tuberculosis, multiple concurrent infectious sites, or prolonged systemic corticosteroid use exceeding a prednisolone-equivalent dose of 1 mg/kg/day for more than one month.

The main study variables were NMLR and SII. Clinical diagnosis (PTB or CAP) was used as the grouping variable for comparative analyses and as an independent variable in the exploratory regression analyses. Potential confounding variables included age, sex, smoking history, and comorbidities. Smoking history was categorized according to documented active or passive smoking exposure, while comorbidity status was categorized according to the presence or absence of documented coexisting diseases at diagnosis. Baseline laboratory data were obtained from the first complete blood count performed before initiation of anti-tuberculosis or antimicrobial treatment.

NMLR was calculated by dividing the absolute neutrophil count by the sum of the absolute monocyte and lymphocyte counts [3]:

$NMLR = \frac{\text{absolute neutrophil count}}{(\text{absolute monocyte count} + \text{absolute lymphocyte count})}$

SII was calculated as the product of the absolute neutrophil and platelet counts divided by the absolute lymphocyte count [13]:

$SII = \frac{(\text{absolute neutrophil count} \times \text{absolute platelet count})}{(\text{absolute lymphocyte count})}$

All data were recorded using a standardized data collection form.

Statistical analyses were performed using IBM SPSS Statistics for Windows version 27.0 (IBM Corp., Armonk, NY). Continuous variables were assessed for distribution using the Shapiro-Wilk test and were presented as mean ± standard deviation for normally distributed data or median with minimum-maximum values for non-normally distributed data. Categorical variables were presented as frequencies and percentages. Because NMLR and SII were not normally distributed, comparisons between the PTB and CAP groups were performed using the Mann-Whitney U test. Categorical variables were compared using the chi-square test with continuity correction or Fisher's exact test, as appropriate.

As secondary exploratory analyses, binary logistic regression was performed with dichotomized NMLR or SII as the dependent variable (≤cutoff = 0; >cutoff = 1), using a backward stepwise likelihood-ratio procedure. Age was entered as a continuous predictor, whereas diagnosis (PTB = 0; CAP = 1), sex (female = 0; male = 1), smoking history (no = 0; yes = 1), and comorbidity status (absent = 0; present = 1) were entered as binary predictors. For regression analysis, NMLR and SII were categorized using previously reported literature-based cut-off values (NMLR ≤2.385 vs. >2.385 [5]; SII ≤2128.32 vs. >2128.32 [4]). Additional analyses used sample-derived median cut-off values of 2.819 for NMLR and 1311.08 for SII. The literature-based and sample-derived cut-offs were analyzed separately.

## Results

Study population and subject selection

A total of 49 eligible subjects were enrolled in this cross-sectional study, comprising 25 treatment-naive, bacteriologically confirmed PTB patients and 24 treatment-naive CAP patients admitted to Ngoerah Hospital between January 2022 and September 2025. The baseline demographic and clinical features of the study cohorts are presented in Table 1.

**Table 1:** Demographic and Clinical Characteristics of PTB and CAP Cohorts Data are presented as mean ± standard deviation (SD) or frequency (n) and percentage (%), as appropriate ᵃ Continuity-corrected chi-square test. ᵇ Percentages are calculated among patients with at least one comorbidity; multiple comorbidities could be reported for the same patient. Statistical tests: Independent-samples t-tests were used for age. Continuity-corrected chi-square tests were used for sex and smoking status. Fisher’s exact test was used for the presence of comorbidity. A p-value <0.05 was considered statistically significant. PTB: pulmonary tuberculosis; CAP: community-acquired pneumonia

Characteristics	PTB (n = 25)	CAP (n = 24)	Test statistic	p-value
Age (years)			t = −2.950	0.005
mean ± SD	47.4 ± 15.75	61.0 ± 16.52		
Sex, n (%)			χ² = 3.673ᵃ	0.055
Male	11 (44.0)	18 (75.0)		
Female	14 (56.0)	6 (25.0)		
Smoking status, n (%)			χ² = 4.983ᵃ	0.026
No	14 (56.0)	5 (20.8)		
Yes	11 (44.0)	19 (79.2)		
Comorbidity, n (%)			Fisher’s exact test	0.002
Present	16 (64.0)	24 (100.0)		
Absent	9 (36.0)	0 (0.0)		
Comorbidities n (%)ᵇ				
Hypertension	1 (6.3)	8 (33.3)		
Diabetes mellitus	3 (18.8)	6 (25.0)		
Acute decompensated heart failure	0 (0.0)	7 (29.2)		
Chronic kidney disease	6 (37.5)	0 (0.0)		
Coronary artery disease	0 (0.0)	4 (16.7)		
Stroke	0 (0.0)	4 (16.7)		
Asthma	2 (12.5)	1 (4.2)		
Bronchiectasis	2 (12.5)	1 (4.2)		
Congestive heart failure	1 (6.3)	2 (8.3)		
Systemic lupus erythematosus	2 (12.5)	0 (0.0)		
Other	2 (12.5)	4 (16.7)		

The mean age of patients in the CAP group was statistically significantly higher than that of patients in the PTB group (61.0 ± 16.52 years vs. 47.4 ± 15.75 years; p = 0.005).

Distribution by sex showed that the PTB cohort contained 11 (44%) males and 14 (56%) females, whereas the CAP cohort contained 18 (75%) males and 6 (25%) females; this difference did not achieve statistical significance (p = 0.055). A history of active or passive smoking was present in 11 (44%) PTB patients and 19 (79.2%) CAP patients, demonstrating a statistically significant difference between the two groups (p = 0.026).

Comorbid conditions were identified in 16 (64%) PTB patients and in 24 (100%) CAP patients, showing a statistically significantly higher prevalence in the CAP cohort (p = 0.002). Among patients with comorbidities, the predominant condition in the PTB cohort was chronic kidney disease (CKD; 37.5%, n = 6/16), followed by diabetes mellitus (DM; 18.8%, n = 3/16), asthma (12.5%, n = 2/16), bronchiectasis (12.5%, n = 2/16), and systemic lupus erythematosus (SLE; 12.5%, n = 2/16). In the CAP cohort, the most frequent comorbidities were hypertension (33.3%, n = 8/24), acute decompensated heart failure (ADHF; 29.2%, n = 7/24), DM (25%, n = 6/24), coronary artery disease (CAD; 16.7%, n = 4/24), and stroke (16.7%, n = 4/24).

Distribution of laboratory parameters

Shapiro-Wilk normality testing demonstrated non-normal distributions for absolute neutrophil count (PTB p = 0.005; CAP p = 0.005), absolute lymphocyte count (PTB p = 0.001; CAP p < 0.001), and absolute monocyte count (PTB p = 0.002; CAP p < 0.001). Platelet counts followed a normal distribution in both PTB (p = 0.604) and CAP (p = 0.668) cohorts.

The median absolute neutrophil count was 4.95 x 10³/µL (range: 2.43-10.60 x 10³/µL) in PTB and 8.44 x 10³/µL (range: 2.90-22.78 x 10³/µL) in CAP. Median absolute lymphocyte counts were 1.24 x 10³/µL (range: 0.44-4.51 x 10³/µL) in PTB and 1.49 x 10³/µL (range: 0.21-4.43 x 10³/µL) in CAP. Median absolute monocyte counts were 0.49 x 10³/µL (range: 0.19-1.69 x 10³/µL) in PTB and 0.86 x 10³/µL (range: 0.22-1.74 x 10³/µL) in CAP. Mean platelet counts were 318.56 ± 109.393 x 10³/µL in PTB and 260.38 ± 75.725 x 10³/µL in CAP (Table 2).

**Table 2:** Primary Laboratory Parameters Between PTB and CAP Data are presented as median (minimum-maximum) for non-normally distributed continuous variables and mean ± standard deviation (SD) for normally distributed continuous variables. Normality was assessed using the Shapiro-Wilk test. PTB: pulmonary tuberculosis; CAP: community-acquired pneumonia

Parameters	PTB (n = 25)	CAP (n = 24)
Absolute neutrophils (x10³/µL)	4.95 (2.43-10.60)	8.44 (2.90-22.78)
Absolute lymphocytes (x10³/µL)	1.24 (0.44-4.51)	1.49 (0.21-4.43)
Absolute monocytes (x10³/µL)	0.49 (0.19-1.69)	0.86 (0.22-1.74)
Platelets (x10³/µL)	318.56 ± 109.393	260.38 ± 75.725

Comparison of NMLR between PTB and CAP

Shapiro-Wilk testing confirmed non-normal distributions for pre-treatment NMLR values in both PTB (p = 0.027) and CAP (p < 0.001) groups. Bivariate evaluation using the Mann-Whitney U test revealed a median NMLR of 2.63 (range: 0.97-6.73) in the PTB group and 3.26 (range: 1.11-18.83) in the CAP group. This numerical difference was not statistically significant (Mann-Whitney U = 213.000, Z = -1.740, p = 0.082).

Comparison of SII between PTB and CAP

Shapiro-Wilk testing confirmed non-normal distributions for pre-treatment SII values in both PTB (p < 0.001) and CAP (p < 0.001) groups. Mann-Whitney U test comparison demonstrated a median SII of 1241.45 (range: 240.17-5,868.25) in the PTB group and 1,335.77 (range: 367.40-11,245.37) in the CAP group. This difference was not statistically significant (Mann-Whitney U = 246.000, Z = -1.080, p = 0.280).

Categorical comparisons

Categorical comparisons of NMLR and SII using predefined threshold values are detailed in Table 3. When applying the literature-derived NMLR cutoff of > 2.385, 15 (60%) PTB patients and 18 (75%) CAP patients had values above the threshold (p = 0.415). Applying the sample median NMLR cutoff of > 2.819 showed that 9 (36%) PTB patients and 15 (62.5%) CAP patients exceeded the cutoff (p = 0.117). When applying the literature-derived SII cutoff of > 2,128.32, 6 (24%) PTB patients and 8 (33.3%) CAP patients had values above the cutoff (p = 0.684). Applying the sample median SII cutoff of > 1,311.08 revealed that 12 (48%) PTB patients and 12 (50%) CAP patients exceeded the threshold (p = 1.000).

**Table 3:** Inflammatory Ratios, and Categorical Threshold Comparisons Between PTB and CAP Continuous variables are presented as median (minimum-maximum), and categorical variables are presented as frequency (n) and percentage (%). Continuous NMLR and SII values were compared between the PTB and CAP groups using the Mann-Whitney U test and are reported with the U and Z statistics. Categorical threshold comparisons were performed using the chi-square test with continuity correction and are reported with the χ² statistic. A two-sided p-value <0.05 was considered statistically significant. PTB = pulmonary tuberculosis; CAP = community-acquired pneumonia; NMLR = neutrophil-to-monocyte-plus-lymphocyte ratio; SII = systemic immune-inflammation index.

Parameter / Variable	PTB (n = 25)	CAP (n = 24)	Test Statistic	p-value
Continuous inflammatory indices				
NMLR, median (min-max)	2.63 (0.97-6.73)	3.26 (1.11-18.83)	U = 213.000; Z = -1.740	0.082
SII, median (min-max)	1241.45 (240.17-5,868.25)	1,335.77 (367.40-11,245.37)	U = 246.000; Z = -1.080	0.28
Categorical threshold comparisons, n (%)				
NMLR>2.385 (literature cutoff)	15 (60.0)	18 (75.0)	χ² = 0.664	0.415
NMLR>2.819 (sample median cutoff)	9 (36.0)	15 (62.5)	χ² = 2.462	0.117
SII >2,128.32 (literature cutoff)	6 (24.0)	8 (33.3)	χ² = 0.165	0.684
SII >1,311.08 (sample median cutoff)	12 (48.0)	12 (50.0)	χ² = 0.000	1

Multivariable analysis

Multivariable binary logistic regression models adjusting for age, sex, smoking status, and comorbidity status are summarized in Table 4.

**Table 4:** Multivariable Binary Logistic Regression Models for Dichotomized NMLR and SII Parameters Multivariable binary logistic regression was used to evaluate the association between dichotomized NMLR or SII and clinical diagnosis while adjusting for age, sex, smoking history, and comorbidity status. Regression parameters are presented as B (regression coefficient), SE (standard error), Wald (Wald chi-square statistic), p-value, odds ratio (OR/ Exp(B)), and 95% confidence interval (CI) for the OR. A two-sided p-value <0.05 was considered statistically significant. NMLR = neutrophil-to-monocyte-plus-lymphocyte ratio; SII = systemic immune-inflammation index; B = regression coefficient; SE = standard error; OR = odds ratio; CI = confidence interval

Model / Predictor	B	SE	Wald	p-value	OR / Exp(B)	95% CI for OR
Model 1: NMLR Cutoff > 2.385 (Nagelkerke R^2^ = 0.178)						
Comorbidities	-2.331	1.174	3.939	0.047	0.097	0.010-0.971
Diagnosis	1.35	0.69	3.827	0.0504	3.86	0.997-14.916
Constant	2.079	1.061	3.844	0.0499	8	-
Model 2: NMLR Cutoff > 2.819 (Nagelkerke R^2^ = 0.091)						
Diagnosis	1.086	0.593	3.358	0.067	2.96	0.927-9.469
Constant	-0.575	0.417	1.907	0.167	0.563	-
Model 3: SII Cutoff > 2128.32 (Nagelkerke R^2^= 0.000)						
Constant	-0.916	0.316	8.396	0.004	0.4	-
Model 4: SII Cutoff > 1311.08 (Nagelkerke R^2^ = 0.000)						
Constant	-0.041	0.286	0.02	0.886	0.96	-

In the model evaluating NMLR using the literature cutoff (> 2.385), comorbidity status remained a statistically significant independent variable (B = -2.331, p = 0.047, OR = 0.097, 95% CI: 0.010-0.971). In this model, the diagnosis of CAP relative to PTB demonstrated B = 1.350, p = 0.0504, OR = 3.86 (95% CI: 0.997-14.916), with a constant of B = 2.079 (p = 0.0499) and a Nagelkerke R² of 0.178. In the model evaluating NMLR using the sample median cutoff (> 2.819), diagnosis demonstrated B = 1.086, p = 0.067, OR = 2.96 (95% CI: 0.927-9.469), with a constant of B = -0.575 (p = 0.167) and a Nagelkerke R² of 0.091; no variables achieved statistical significance.

In multivariable regression models for SII, no independent variables (diagnosis, age, sex, smoking status, or comorbidities) retained statistical significance using either the literature cutoff of > 2,128.32 (Constant B = -0.916, p = 0.004, Nagelkerke R² = 0.000) or the sample median cutoff of > 1,311.08 (Constant B = -0.041, p = 0.886, Nagelkerke R² = 0.000).

There were no statistically significant differences in median peripheral blood NMLR (2.63 vs. 3.26; p = 0.082) or SII (1,241.45 vs. 1,335.77; p = 0.280) between patients with treatment-naive pulmonary tuberculosis and treatment-naive community-acquired pneumonia.

## Discussion

In this cross-sectional observational study comparing 25 treatment-naive, bacteriologically confirmed PTB patients and 24 treatment-naive CAP patients, no statistically significant differences in pre-treatment peripheral blood NMLR and SII were detected between the two groups. Although median values for both inflammatory indices were numerically higher in the CAP cohort than in the PTB cohort (NMLR: 3.26 vs. 2.63, p = 0.082; SII: 1,335.77 vs. 1,241.45, p = 0.280), these trends show some distribution overlap and wide variability. In multivariable logistic regression modeling adjusting for age, sex, smoking history, and comorbidity status, diagnosis (CAP vs. PTB) was not an independent predictor of dichotomized NMLR or SII. Instead, comorbidity status emerged as the sole statistically significant variable associated with NMLR when applying a literature-derived cutoff of > 2.385 (B = -2.331, p = 0.047, OR = 0.097, 95% CI: 0.010-0.971).

The observed lack of a statistically significant difference in median NMLR between PTB and CAP cohorts (2.63 vs. 3.26; p = 0.082) diverges from previously published literature. Jeon et al. (2019) demonstrated a significantly higher mean NMLR in non-tuberculous infectious lung diseases compared to PTB (6.80 ± 4.19 vs. 2.71 ± 1.28; p < 0.001) and established a diagnostic cutoff of < 3.95 for discriminating PTB [3]. Similarly, Mohammed et al. (2023) reported significantly elevated NMLR in CAP relative to PTB (p < 0.001) and proposed an NMLR threshold > 2.385 for predicting pneumonia [5]. Aygün et al. (2024) also reported elevated NMLR in pediatric CAP compared to PTB or healthy controls [14]. Furthermore, Tu et al. (2023) observed that NMLR could discriminate PTB from genitourinary TB (AUC = 0.920, p < 0.001), where genitourinary TB exhibited lower ratios due to preserved cellular immunity [15].

The discrepancy between the non-significant findings of this study and earlier research may partly reflect methodological differences, including data variability in the CAP cohort, sample size considerations, and the high burden of chronic comorbidities in a tertiary referral hospital population. One possible biological explanation for these numerical trends, based on previously described immune responses, involves differences in host responses to acute versus chronic respiratory infection. Acute bacterial CAP induces a rapid neutrophilic influx into alveolar spaces driven by CXCL8 (IL-8) chemokine gradients, combined with stress-induced lymphopenia and monocyte recruitment, which may contribute to a higher NMLR. Conversely, PTB is characterized by chronic granulomatous inflammation wherein persistent Mycobacterium tuberculosis within alveolar macrophages stimulates IL-12, Th1 differentiation, IFN-γ release, and continuous monocyte recruitment alongside adaptive lymphocyte activation, potentially contributing to a lower NMLR relative to acute bacterial infection [3,16,17]. These mechanisms were not directly evaluated in the present study because cytokines and other inflammatory pathway markers were not measured and should therefore be considered biologically plausible explanations rather than mechanisms established by our data.

SII values showed no statistically significant difference between PTB and CAP patients (median 1,241.45 vs. 1,335.77; p = 0.280). The median SII observed in the PTB cohort aligns closely with the cutoff reported by He et al. (2024) for cavitary PTB (1,252.045), but remains below the threshold reported by Yu et al. (2024) for active PTB versus non-TB lung disease (2,128.32) [4,18]. In CAP literature, Tümüklü and Felek (2025) reported a mean SII of 1,301.28 ± 886.78 in CAP patients compared to 545.20 in healthy controls, which is consistent with the median SII observed in the present CAP cohort [16]. However, higher SII cutoffs have been reported for predicting CAP severity and mortality, including > 3,551 by Acar et al. (2021), > 4,689.82 by Chen et al. (2025), and 114.72 for general emergency department CAP mortality by Tortum et al. (2023) [10,19,20].

SII integrates absolute neutrophils, platelets, and lymphocytes to reflect systemic inflammatory equilibrium. Based on previously described mechanisms, acute CAP may be accompanied by neutrophilia and stress-induced lymphopenia, while activated platelets participate in inflammatory and endothelial responses. In PTB, persistent mycobacterial infection may contribute to reactive thrombocytosis through IL-6-mediated thrombopoiesis alongside chronic cellular immune dysregulation. These mechanisms were not directly measured in the present study and are discussed only as possible biological explanations for the observed findings. The lack of statistical separation between PTB and CAP in this study may partly reflect the wide variability in the CAP cohort, including an SII value reaching 11,245.37, together with baseline inflammatory elevation from co-existing chronic diseases and the high smoking prevalence in CAP patients [10,13,16].

Demographic and clinical profiles differed substantially between cohorts and may have influenced baseline systemic inflammatory parameters. CAP patients were significantly older than PTB patients (mean age 61.0 ± 16.52 years vs. 47.4 ± 15.75 years; p = 0.005), consistent with age-related immunosenescence and chronic disease accumulation. A history of active or passive smoking was significantly more frequent in CAP patients than in PTB patients (79.2% vs. 44%; p = 0.026). Chronic exposure to tobacco smoke damages mucociliary clearance, activates alveolar macrophages, and stimulates pro-inflammatory cytokine release (TNF-α, IL-1β, IL-6), promoting peripheral neutrophilia and elevated baseline SII [16].

Comorbidity prevalence was 100% in the CAP cohort compared to 64% in the PTB cohort (p = 0.002). Among PTB patients, chronic kidney disease (CKD; 37.5%) and diabetes mellitus (DM; 18.8%) predominated, whereas CAP patients presented primarily with hypertension (33.3%), acute decompensated heart failure (ADHF; 29.2%), DM (25%), coronary artery disease (16.7%), and stroke (16.7%). Chronic conditions such as heart failure and CKD are associated with persistent low-grade systemic inflammation and altered leukocyte kinetics, which may contribute to overlapping hematological indices and potentially obscure pathogen-specific host immune responses [7].

Multivariable binary logistic regression was executed to adjust for confounding by age, sex, smoking history, and comorbidity status. In the model evaluating NMLR using the literature cutoff (> 2.385), comorbidity status was the only variable significantly associated with NMLR (B = -2.331, p = 0.047, OR = 0.097, 95% CI: 0.010-0.971). The negative B coefficient indicates that patients with comorbidities were statistically more likely to have NMLR ≤ 2.385. Diagnosis (CAP vs. PTB) approached but did not achieve statistical significance (B = 1.350, p = 0.0504, OR = 3.86, 95% CI: 0.997-14.916), with the model explaining 17.8% of variance (Nagelkerke R² = 0.178) [5]. When evaluating NMLR using the sample median cutoff (> 2.819), diagnosis was not statistically significant (B = 1.086, p = 0.067, OR = 2.96, 95% CI: 0.927-9.469, Nagelkerke R² = 0.091). For SII, no independent variables maintained statistical significance using either literature (> 2,128.32) or median (> 1,311.08) cutoffs (Nagelkerke R² = 0.000) [4]. These models reflect statistical associations rather than direct causal relationships. Given the limited number of participants, these exploratory multivariable findings should be interpreted cautiously and should not be considered evidence of diagnostic validation.

NMLR and SII are calculated from routine complete blood count (CBC) testing, making them low-cost, rapidly available, and easily accessible parameters in clinical practice. In the present cohort, however, neither index showed a statistically significant difference between PTB and CAP, suggesting limited discriminatory ability when used alone in this specific population. These findings should not be interpreted as establishing a lack of diagnostic utility in other populations. Rather, NMLR and SII may provide complementary information regarding systemic immune-inflammatory responses, while clinical differentiation between PTB and CAP should continue to rely on appropriate microbiological, clinical, and radiological assessment.

Several limitations of this study should be acknowledged. First, although the study achieved the prespecified minimum sample size, the number of participants remained relatively limited, particularly for exploratory multivariable analyses, and the single-center setting at a tertiary referral hospital may limit the generalizability of the findings to other healthcare settings and patient populations. Second, the retrospective use of medical records and consecutive inclusion of eligible patients may introduce selection bias and limit control over potentially relevant unmeasured variables. Third, substantial baseline differences were present between the PTB and CAP cohorts, particularly in age, smoking history, and comorbidity burden. Although multivariable analyses were performed to account for these factors, residual confounding cannot be excluded. The particularly high prevalence of chronic comorbidities in the CAP group (100% vs. 64% in the PTB group) may also have influenced the inflammatory indices and obscured infection-specific differences. Finally, the sample-derived median cutoffs were used for exploratory analyses and should not be interpreted as validated diagnostic thresholds. Accordingly, the absence of statistically significant differences in this cohort should not be interpreted as evidence that NMLR and SII lack diagnostic value in other populations.

The strengths of this study include the direct comparison of treatment-naive PTB and CAP patients in a real-world clinical setting; strict diagnostic criteria, including bacteriological confirmation of PTB and clinical and radiological criteria for CAP independently verified by attending pulmonologists before therapy; inclusion of a tertiary care population with chronic comorbidities; and the use of non-parametric testing and exploratory multivariable logistic regression. These findings also identify directions for future research. Larger prospective multicenter studies are needed to further evaluate these hematological parameters across different healthcare settings. Future studies should incorporate explicit control, subgroup matching, or statistical stratification for chronic comorbidities such as CKD and heart failure and should evaluate multiparametric diagnostic models combining hematological, biochemical, and clinical indicators rather than relying on single inflammatory ratios.

## Conclusions

This retrospective, cross-sectional study found no statistically significant differences in pre-treatment NMLR and SII between patients with PTB and CAP. In this cohort, these findings suggest that NMLR and SII alone may have limited ability to distinguish between PTB and CAP and should not replace definitive microbiological confirmation supported by clinical and radiological assessment. Given their rapid availability and low cost, these indices may still provide complementary information regarding systemic immune-inflammatory responses. However, these findings should be interpreted cautiously considering the relatively limited number of participants, single-center retrospective design, baseline differences between the groups, and high comorbidity burden. The exploratory cutoff-based and multivariable findings require validation in larger prospective multicenter studies before their diagnostic utility can be established.
